# Assessment of the Health Impacts of Climate Change in Kiribati

**DOI:** 10.3390/ijerph110505224

**Published:** 2014-05-14

**Authors:** Lachlan McIver, Alistair Woodward, Seren Davies, Tebikau Tibwe, Steven Iddings

**Affiliations:** 1 Australian National University, Canberra, ACT 0200, Australia; 2 Division of Pacific Technical Support, World Health Organization, Suva, Fiji; E-Mail: iddingss@wpro.who.int; 3 School of Population Health, Faculty of Medical and Health Sciences, University of Auckland, Auckland 1010, New Zealand; E-Mail: a.woodward@auckland.ac.nz; 4 Ministry of Health and Medical Services, Bikenibau, Republic of Kiribati; E-Mails: senyda@gmail.com (S.D.); tnoran@gmail.com (T.T.)

**Keywords:** climate change, health, adaptation, small island state

## Abstract

Kiribati—a low-lying, resource-poor Pacific atoll nation—is one of the most vulnerable countries in the World to the impacts of climate change, including the likely detrimental effects on human health. We describe the preparation of a climate change and health adaptation plan for Kiribati carried out by the World Health Organization and the Kiribati Ministry of Health and Medical Services, including an assessment of risks to health, sources of vulnerability and suggestions for highest priority adaptation responses. This paper identifies advantages and disadvantages in the process that was followed, lays out a future direction of climate change and health adaptation work in Kiribati, and proposes lessons that may be applicable to other small, developing island nations as they prepare for and adapt to the impacts of climate change on health.

## 1. Introduction

### 1.1. Background to Climate Change in Kiribati

**Figure 1:**
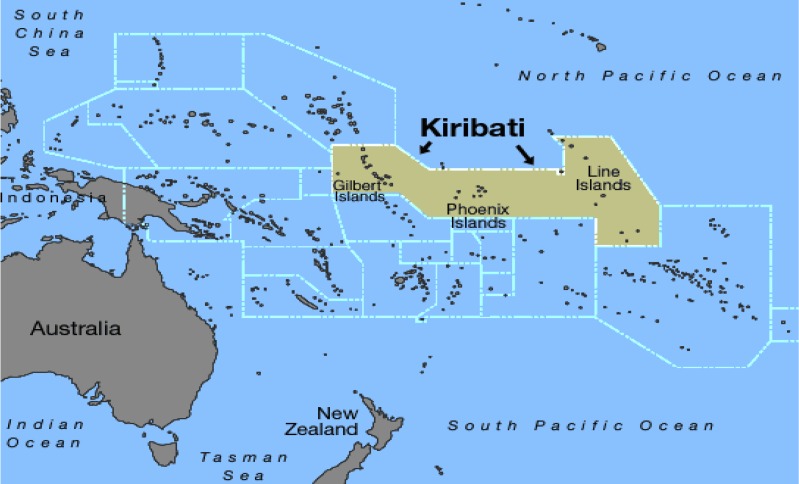
Map of Kiribati (source: Office of Te Beretitenti, Government of Kiribati).

The Republic of Kiribati (Kiribati) is a low-lying country of thirty-three atolls, straddling the equator in the central Pacific (see Figure 1), with an average elevation of less than three metres above sea level. Roughly half of the total population of approximately 105,000 reside on the small atoll of South Tarawa (area 16 km^2^), with population densities approaching 10,000 persons per square kilometre in the most crowded parts of the atoll [1].

The climate of Kiribati is hot and humid, with very little variation in maximum and minimum temperatures throughout the year. Droughts, usually associated with La Niña, can be very severe in Kiribati, such as the events that occurred in 1988–1989, 1998–1999, 2007–2009 and early 2011.

Kiribati is highly vulnerable to the impacts of climate change, due to, *inter alia*, limited land area and opportunities for domestic agriculture, over-crowding, the low elevation of the islands and the lack of safe and secure supplies of potable water. The particular threats posed by climate change for Kiribati include sea-level rise, increasing air and sea-surface temperatures, ocean acidification, altered rainfall patterns and the unpredictability of events such as droughts, storm surges and extreme high winds [2]. On short time scales, some of these changes can be seen already: ambient air temperatures (closely related to sea-surface temperatures in the case of Kiribati) have increased by approximately one degree Celsius since 1950 [2], and since 1992 sea-level around Kiribati has risen by 3.9 mm per year, three times faster than the global average [3].

### 1.2. Rationale for Assessment of Climate Change and Health in Kiribati

Climate change threatens not only the land and livelihoods of *i-Kiribati* communities, but also the health of the population. The pathways linking climate change to human health have been described extensively in the literature [4,5,6,7,8]; however relatively little of this work to date has focussed on the South Pacific [9,10,11,12,13].

From 2010 to 2012, the World Health Organization (WHO) Division of Pacific Technical Support undertook a project supporting eleven Pacific island countries—including Kiribati—assess their vulnerability to climate change and compile national health adaptation strategies to manage those risks to health. The mandates for this project included the 2009 *Madang Commitment*, in which the Health Ministers of Pacific island countries committed to action on climate change [14]; and the 2008 WHO Regional Framework on climate change and health [15].

**Figure 2:**
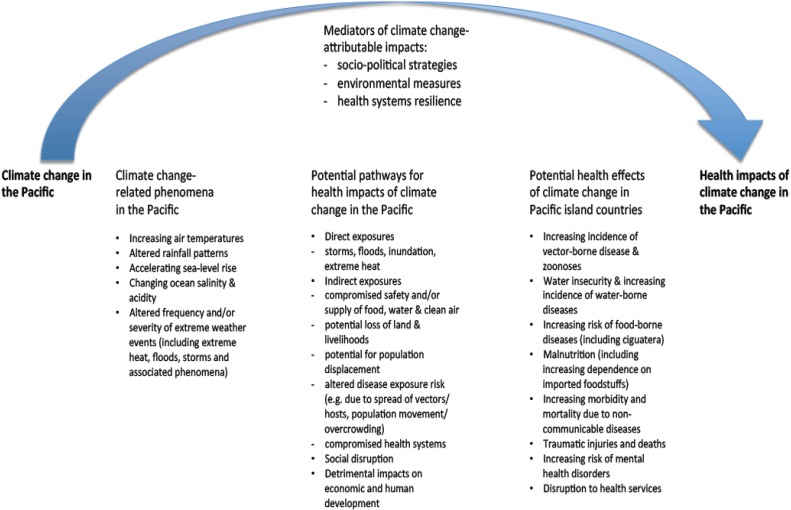
Overview of climate change and health vulnerabilities in Pacific island countries.

The main climate change-related health risks of concern in Pacific island countries, as identified in the WHO-supported vulnerability and adaptation assessment project, are summarised in Figure 2.

Recognising the vulnerability of the country to climate change, Kiribati was one of the first Pacific island states to prepare a National Adaptation Programme of Action (NAPA), prior to the commencement of the WHO-supported climate change and health project. Kiribati’s NAPA, finalised in 2007, made reference to several possible health impacts of climate change, including diarrhoeal disease, dengue fever, fish poisoning, social disruption and the health impacts of extreme weather events [16].

Following on from the NAPA, WHO and the Kiribati Ministry of Health and Medical Services (MHMS) undertook a more rigorous climate change and health vulnerability assessment. This led to the development of a National Climate Change and Health Action Plan (NCCHAP), as the health sector’s formal contribution to the cross-sectoral national adaptation planning process in Kiribati.

This paper describes the methods employed in Kiribati’s climate change and health vulnerability assessment and adaptation planning, and summarises the outcomes.

It is important to note that the contemporary literature on health vulnerability and adaptation assessments includes descriptions of approaches to assessing climate change and health vulnerabilities, of both quantitative [17,18] and qualitative [19] varieties, but these methods do not always reflect the practical necessities of climate change and health work in small, developing countries, where resources are scant and the relevant data is scarce. We describe a “middle way” for climate change and health vulnerability and adaptation assessments, one that better fits, we suggest, the circumstances of small island developing states.

## 2. Methodology

**Figure 3:**
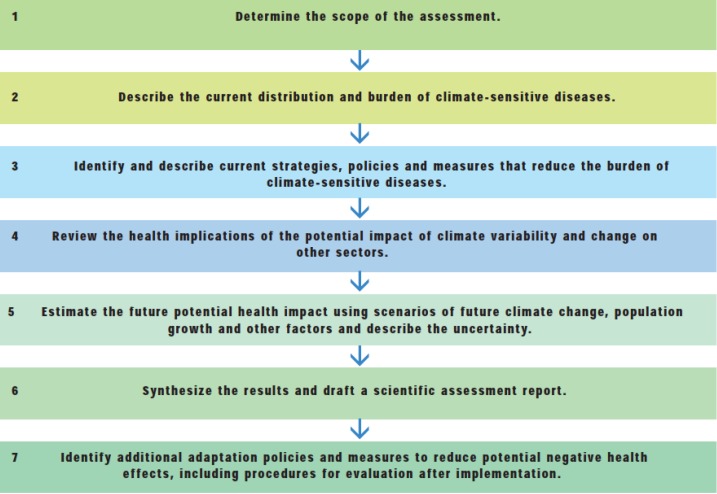
Steps in assessing vulnerability and adaptation (Source: Kovats *et al.*, 2003 [20]).

The approaches that have been recommended for carrying out climate change and health vulnerability assessments and adaptation planning typically include a number of steps, from identifying the current burden of climate-sensitive diseases, to estimation of the future climate-change attributable burden of diseases, with consideration of strategies to minimize climate change-related risks to health and acknowledgement of the health impacts of adaptation in other sectors. The process employed in the vulnerability and adaptation assessment in Kiribati is detailed below, with reference to the guidelines provided in the literature [18,20] and summarized in Figure 3.

### 2.1. Determining the Scope of the Assessment

In Kiribati, the climate change and health vulnerability assessment began with a review of the available data on climate and climate-sensitive diseases, along with consideration of current public health capacity and adaptation activities. The WHO-MHMS team undertook a series of consultations with stakeholders across departments of the MHMS, as well as other government agencies and non-government organizations (see Table 1), seeking views on the key problems posed by climate change, risks to health and priority adaptation strategies and activities for Kiribati.

The assessment began with an inception meeting (of representatives from Kiribati, Tuvalu, Niue, Tonga and the Cook Islands) in Auckland in 2010, and spanned four visits to Kiribati by WHO consultants and staff through 2011 and 2012.

**Table 1:** Matrix used to assess climate-sensitive health risks in Kiribati, in terms of their likelihood and impact.

Likelihood	Impact (Considering Consequence and Coping Capacity)				
	Insignificant	Minor	Moderate	Major	Catastrophic
Almost Certain	Medium	Medium	High	Extreme	Extreme
Likely	Low	Medium	High	High	Extreme
Possible	Low	Medium	Medium	High	High
Unlikely	Low	Low	Medium	Medium	Medium
Rare	Low	Low	Low	Low	Medium

### 2.2. Describing the Current Distribution and Burden of Climate-Sensitive Diseases

The WHO-MHMS team considered a long list of health issues in Kiribati that have the potential to be affected by changes in climate. In doing so, the team consulted with health stakeholders from a number of fields (environmental health, communicable diseases, NCDs, nutrition and mental health), including the relevant heads of departments within the MHMS. The list of potential climate-sensitive health risks generated for Kiribati included a number of communicable diseases (water-, food- and vector-borne diseases, infections of the eyes, skin and respiratory tract and zoonotic diseases), injuries, non-communicable diseases (NCDs) and heat-related illness, food security, malnutrition and mental health. Despite the lack of detailed information on disease rates, it was possible to identify the most pressing climate-sensitive health problems in Kiribati via consultation and review of the major causes of morbidity and mortality from routinely-collected MHMS data.

In order to prioritize adaptation strategies, in the absence of detailed information on risks and trends, the process in Kiribati relied to a significant extent on expert judgement. This approach employed estimates of the likelihood that climate change would exacerbate particular health risks, alongside the potential impact of these increased risks on the burden of disease. This “likelihood *versus* impact” model has proved useful in climate change and health impact assessments elsewhere in the Pacific [13] and is elaborated in Table 1. The core WHO-MHMS climate change and health team carried out this subjective analysis; on the advice of the MHMS stakeholders, the process was further simplified by splitting the climate-sensitive health risks into two categories only: “high risk” and “low risk” (see Results below).

### 2.3. Identifying and Describing Current Strategies, Policies and Measures that Reduce the Burden of Climate-Sensitive Diseases

To determine the priorities for this plan, the team focused on conditions that were strongly linked to changes in climate, that would add substantially to the burden of disease in Kiribati, and were tractable—*i.e.*, where evidence was available on interventions that were likely to alleviate the current and future burdens of these diseases, for example water, sanitation and hygiene interventions for diarrhoeal illness [21]. While the MHMS had in place some routine policies and measures related to management of these risks—for example the National Water Resources and Sanitation Policies and the annual workplan of the MHMS Environmental Health Unit (EHU)—it was clear from the simple needs analysis conducted during the assessment process that substantial shortfalls in capacity and resources to manage these risks remained.

### 2.4. Reviewing the Health Implications of the Potential Impact of Climate Variability and Change on other Sectors

It was evident that, despite mention of health vulnerabilities in the NAPA, the current suite of adaptation plans and activities in Kiribati had not sufficiently taken into account the health implications of climate change. In particular, the marquee national adaptation program—the Kiribati Adaptation Plan (KAP), then approaching its third phase—was focused on infrastructure and coastal protection (e.g., construction of seawalls and planting of mangroves) without explicit consideration of the potential health impacts of these measures. Nor did the KAP grasp the opportunities—the health “co-benefits”—that might be achieved by emphasizing adaptation strategies closely aligned with health [22,23,24].

### 2.5. Estimating the Future Potential Health Impact Using Scenarios of Future Climate Change, Population Growth and Other Factors, and Describing the Related Uncertainty

It was not feasible, given the lack of reliable historical health data and down-scaled climate projections, to calculate precisely the future climate-change attributable burdens of disease. However, on the basis of this assessment and the international literature, it was deemed highly likely that climate change would amplify current health risks. Of particular concern in Kiribati is the potential for climate change effects—particularly sea-level rise—to exacerbate overcrowding and add to the risk of infectious disease transmission [25].

### 2.6. Synthesizing the Results and Drafting a Scientific Assessment Report

The WHO-MHMS team compiled the aforementioned NCCHAP for Kiribati, which synthesized the process, outcomes and recommendations of the vulnerability and adaptation assessment. The most important elements of the plan are included in the Section Results. This NCCHAP has been used to inform funded adaptation project work related to water security and water-borne diseases in Kiribati (see Discussion for further details), and will be incorporated into a regional synthesis report on climate change and health in Pacific island countries, to be launched by WHO later this year.

### 2.7. Identifying Additional Adaptation Policies and Measures to Reduce Potential Negative Health Effects, Including Procedures for Evaluation Following Implementation

Procedures for review of priorities, implementation of plans and evaluation of processes articulated in the NCCHAP were incorporated into the recommendations. It will be a sign of success if future assessments of climate change and health in Kiribati review the NCCHAP methodology, make changes where these are warranted, and update national plans accordingly.

Sources of information considered in the vulnerability assessment and adaptation planning process in Kiribati are summarized in Table 2 below.

**Table 2:** Information considered in the Kiribati climate change and health vulnerability assessment.

Type/Source of Information Reviewed and Consulted	Name/Description of Source and Information Obtained
Data	notifiable disease surveillance data from MHMS (Health Information Unit) annual reports from MHMS historical climate data from Kiribati Meteorology Service climate change data (historical trends and predictions) from Pacific Climate Change Science Program quality of household sanitation and water supplies, information obtained from the 2010 national census population data (2010 census)
Stakeholders	Office of Te Beretitenti (Office of the President) Ministry of Health and Medical Services Ministry of Public Works and Utilities Ministry of Internal and Social Affairs Ministry of Fisheries and Marine Resources Development Ministry of Environment, Lands and Agricultural Development Ministry of Commerce, Industries and Cooperatives Kiribati Port Authority Overseas Environmental Cooperation Centre Secretariat for the Pacific Community (including the South Pacific Applied Geosciences Commission) Kiribati Association of Non-Government Organizations Community members (during health promotion workshops) World Health Organization
Previous/current activities related to climate change adaptation	National Adaptation Programme of Action (NAPA) Kiribati Adaptations Plans (I-III) Climate Change Adaptation Plan and Strategy National Framework for Climate Change Adaptation Second National Communication (to the United Nations Framework Convention on Climate Change) Kiribati Development Plan Kiribati National Development Strategies
Previous/current activities related to public health	Ministry of Health and Medical Services’ Strategic Plan National Population Policy National Disaster Risk Management Plan National Sanitation Implementation Plan

## 3. Results

In determining the priorities for the Kiribati NCCHAP, the WHO-MHMS team focused on conditions that:

(a) have been shown to be strongly linked to changes in climate (based on empirical epidemiological evidence and expert judgement);
(b) would likely add substantially to the burden of disease in Kiribati; and
(c) could be reduced by feasible public and environmental health interventions.

The list of climate-sensitive health risks considered in the final NCCHAP for Kiribati includes is shown in Table 3. In this Table, “low priority” does not necessarily mean unimportant, but rather that the issue in question is considered to be of a relatively lower priority than those deemed to be “high”. Note also the inclusion of disease surveillance, which is clearly not a health condition, but a health-protective measure—this was included because the team judged that surveillance would be central to managing most climate-related risks to health in Kiribati.

**Table 3:** Climate change and health adaptation priorities in Kiribati.

Health Issue Likely to be Affected by Climate Change	Priority
Water safety and water-borne diseases	High
Food safety and food-borne diseases	High
Vector-borne diseases	High
Disease surveillance	High
Respiratory diseases	Low
Malnutrition	Low
Non-communicable diseases and heat-related illness	Low
Ciguatera	Low
Mental health	Low
Reproductive health	Low

The priority areas for climate change and health adaptation in Kiribati identified in this project and listed in Table 3 all meet the abovementioned three criteria of being linked to climate change; likely to exacerbate existing burdens of diseases; and amenable to public and/or environmental health intervention.

### 3.1. Water Safety and Water-borne Diseases

**Figure 4:**
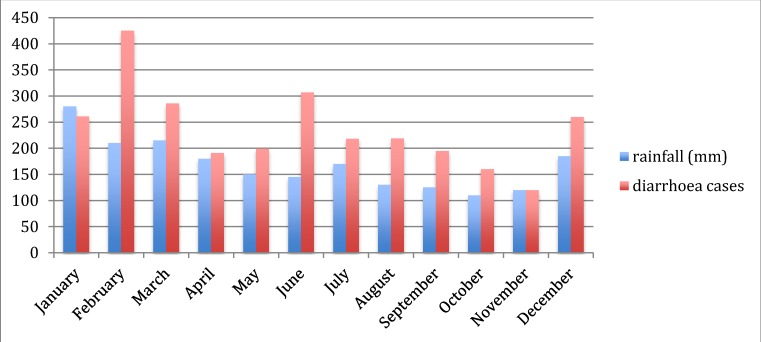
Average monthly rainfall (Kiribati), and reported cases of diarrhoea (all ages) in Betio district, South Tarawa, 2009–2010.

As an atoll country, Kiribati’s potable water is drawn exclusively from aquifers and harvested rainwater. According to a 2011 report compiled by the United Nations Office for the Coordination of Humanitarian Affairs, in collaboration with UNICEF and the Secretariat for the Pacific Community (SPC), distribution, contamination of the aquifers and other effects of population growth and increasing density of settlement all pose challenges to water quality in Kiribati. The same report describes the risks to health and livelihoods posed by drought. Extremes of rainfall are correlated with the incidence of water-borne diseases (such as diarrhoeal disease, cholera and typhoid fever) in the Pacific region [11] and elsewhere [26,27,28,29,30]. In South Tarawa, on the basis of the scant data available, there appeared to be at least a modest seasonal pattern of diarrhoeal disease incidence, with the number of monthly cases of diarrhoea in the heavily populated area of Betio rising with the onset of heavy rains in December in recent years (Figure 4).

Community stakeholders pointed out that end-of-year cultural gatherings of large groups may contribute to an increase in food-borne infections causing diarrhoea, but it is also plausible that run-off following heavy rains and contamination of drinking water sources contributes to this problem. Projections for future climates in Kiribati generally indicate an increase in the days of very heavy rainfall by 2050 [2], raising the possibility of an increased risk of water-borne diseases in heavily contaminated, crowded areas, unless measures are taken to protect water supplies and block the transmission of infections. Cost-effective interventions may include hand-washing, household water treatment and the implementation of water safety plans [31,32,33], ideally in combination with larger-scale improvements to water safety testing and infrastructure where there is a clear need (as there is in Kiribati).

### 3.2. Food Safety and Food-borne Diseases

As an equatorial country which experiences consistently high ambient temperatures and high humidity, with limited facilities for refrigeration and secure food storage, Kiribati is at high risk of illnesses due to contamination of food by bacteria, viruses and toxins. These illnesses may be exacerbated by climate change [34,35,36], but there are opportunities also to reduce risks by improvements in storage, food preparation and handling.

### 3.3. Vector-borne Diseases

Many vector-borne diseases, including those affecting the population of Kiribati (e.g., dengue fever and lymphatic filariasis) are sensitive to changes in climate variables such as temperature, rainfall and humidity [37,38,39,40], and therefore may be influenced by climate change [41]. Kiribati has experienced at least three major epidemics of dengue fever since 2003. There is a competent vector present in the form of the *Aedes aegypti* mosquito, the atolls provide an abundance of breeding sites (including tyres, cans, bottles, shells and coconut husks) and there are high densities of potential human hosts in certain dengue fever “hotspots” on South Tarawa. Improved disease surveillance (e.g., implementation of a syndromic surveillance approach) and mosquito control programs have reduced the frequency and intensity of dengue fever outbreaks in other developing countries [42].

### 3.4. Disease Surveillance

Expanded or enhanced surveillance for climate-sensitive communicable diseases is one of the most frequently-cited examples of climate change adaptation in the health context [43], particularly with respect to climate-based epidemic early warning systems for climate-sensitive diseases such as those listed above [44,45,46].

### 3.5. Respiratory Diseases

Respiratory diseases—including those with infectious causes (e.g., pneumonia, viruses) and other aetiologies such as asthma—have been linked with climate variability and change [47,48]. It is relevant to note that Kiribati has very high rates of smoking and overcrowding, which are additional risk factors for transmission of respiratory infections, but are also obvious areas for action in the adaptation context.

### 3.6. Malnutrition

*I-Kiribati* communities, particularly children, are already at significant risk of malnutrition due to the lack of suitable land for agriculture and the country’s increasing dependence on energy-dense, imported foodstuffs. The concern is that climate change will exacerbate this risk via its detrimental effects on crops and fisheries, increasing the incidence of childhood diseases such as diarrhoea (for which malnutrition is a risk factor) and decreasing individuals’ willingness and ability to perform outdoor work or exercise in higher temperatures [5,49]. Much work and investment will be needed to secure suitable nutrition for Kiribati’s population as part of adaptation in the face of climate change. One example is the current attempt to breed drought- and salt-resistant crops such as taro and cassava—staple foods in Kiribati and across the Pacific.

### 3.7. Non-communicable Diseases and Heat-related illnesses

Climate change may have far-reaching impacts on non-communicable diseases (such as diabetes and circulatory disease), via complex and as yet poorly understood pathways [50,51]. As a poor, developing nation that already experiences high levels of cardiovascular diseases, cancer and diabetes, Kiribati is unfortunately situated to experience the additional driving force of climate change on non-communicable diseases. Increasing ambient temperatures are likely to increase hospitalizations and deaths of individuals with cardiovascular and respiratory illnesses, as has been demonstrated in other parts of the world, including tropical regions [52,53].

### 3.8. Ciguatera

Ciguatera (a toxidrome caused by ingestion of a dinoflagellate organism which bio-accumulates in the marine food chain) has been linked with sea-surface temperatures and thus with climate change in the Pacific, albeit with somewhat conflicting results [54,55,56]. Kiribati has among the highest reported rates of “fish poisoning” (noting that this does not necessarily or always imply ciguatera) in the Pacific, and local studies by the Ministry of Fisheries and Marine Resources Development suggest that the number and range of ciguatoxic fish may be expanding in reef areas around Kiribati.

### 3.9. Mental Health

Climate change may threaten the mental health of *i-Kiribati* communities as rising seas erode land and livelihoods, threatening the country’s sovereignty and national identity. Unfortunately, there is relatively little published in the scientific literature on the mental health impacts of climate change outside the northern hemisphere and developed southern hemisphere countries such as Australia [57,58,59], although some inferences may be made from the evidence on the mental health consequences of natural disasters [59,60,61]. In addition, in Kiribati very little is known about the present burden of mental ill-health, so it is challenging to identify with confidence the characteristics of these problems and the groups in the community that are, or will be, most affected.

### 3.10. Reproductive Health

One of the major demographic, health, social and development challenges for Kiribati is how to respond to the country’s rapid population growth. High fertility rates (estimated from the 2010 census as 3.8 children per woman), increasing population density in Tarawa, and decreasing habitable land area due to sea-level rise and coastal inundation have already forced the Government of Kiribati to prioritize population control and resettlement (including policies on emigration) in its National Framework for Climate Change Adaptation. While it may be considered by many to be a reasonably effective adaptation strategy, climate change-induced migration itself is likely to have profound health consequences on both source and recipient communities [62].

### 3.11. Vulnerable Groups

It is well established in the climate change and health literature that certain groups are likely to be disproportionately affected by climate change. Such sub-populations include the very young, the elderly, those with disabilities and pre-existing medical conditions, people residing in highly vulnerable areas (which may be considered to include the entire population of Kiribati, given the unique geographic susceptibilities inherent in the width and elevation of exclusively-atoll countries in the context of climate change) and those in certain occupations (fishers, farmers, construction and other outdoor workers) [53].

## 4. Discussion

### 4.1. Innovations and Challenges in the Vulnerability Assessment and Adaptation Planning Process in Kiribati

The climate change and health vulnerability and adaptation assessment in Kiribati combined quantitative elements—utilizing disease surveillance and climate data where possible, to give an indication of climate and climate-sensitive disease trends—with a strong qualitative element, largely carried out via engagement with stakeholders from the MHMS, other government agencies, community representatives and the Kiribati Association of NGOs (KANGO). This mixed-methods approach utilized the key features from published guidelines such as those described in the Section Methodology above, in synergy with a pragmatic, “no regrets” approach—defined as that which “increases the capacity of society to manage climate risks with a view to reduce the vulnerability of households and maintain or increase the opportunities for sustainable development” [63]—which has been recommended for smaller and/or developing countries and weaker health systems [64]. This process also incorporated elements from the Health Impact Assessment (HIA) literature, which has been adapted to the climate change and health context [19,65,66], particularly with respect to the health adaptation opportunities in non-health sectors (e.g., agriculture, energy, transport and infrastructure) and across the governance spectrum (e.g., from regulation and legislation to ecosystem intervention, research, technological innovation and infrastructure development).

The process of prioritizing climate change-related risks to health in Kiribati was hampered by the lack of reliable long-term data on disease incidence and health care utilization. Given the data paucity, priority risks and adaptation strategies were arrived at in large part by a process of consensus building and “expert opinion”, which has obvious limitations [67].

Nevertheless, the authors believe that a mixed-methods approach to climate change and health vulnerability and adaptation assessment, such as that described herein for Kiribati—grounded in theory but flexible in the face of on-the-ground realities and data and resource constraints—is likely to prove the approach most suitable for small island developing states and many least developed countries in the foreseeable future.

### 4.2. Future Direction of Climate Change and Health Adaptation in Kiribati

The key recommendations contained within the Kiribati NCCHAP reflect the priorities listed above, and relate primarily to strengthening climate-sensitive disease surveillance; improving resourcing for the MHMS EHU (particularly for those activities related to water safety, food safety and vector control); increasing the data quality and analytical capacity of the MHMS Health Information Unit; securing the necessary technical and financial support for community and health sector adaptation; and ensuring appropriate policy development in the field of climate change and health—for example, using the NCCHAP as the health sector’s contribution towards ongoing national adaptation planning in Kiribati.

It is hoped that health adaptation to climate change in Kiribati will be guided by the NCCHAP, but it is also anticipated that this vulnerability and assessment process is merely a starting point, which must be revised and updated regularly to incorporate new information, science and evidence, and to reflect current activities and shifting priorities related to public health in Kiribati.

Early signs of progress in implementing the NCCHAP are evident in a European Union-funded project, housed within the MHMS EHU, directed by the Secretariat for the Pacific Community and based on the NCCHAP, which prioritises water safety and water-borne diseases and aims to strengthen the capacity of the EHU with regards to its activities and resources as a critical aspect of adaptation in Kiribati.

## 5. Conclusions

This paper describes a “middle way” for climate change and health vulnerability assessment and adaptation planning in a low-lying small island developing state. This approach, which combines both quantitative and qualitative elements and has as its foundation the empirical framework for climate change and health assessments drawn from the international literature, but that also relies upon a pragmatic and consensual process to ensure relevance and feasibility with respect to adaptation plans and implementation thereof, may be the optimal strategy for other, comparable developing and/or island countries. There are a number of features of climate change and health risk and adaptation planning described in this paper which are not unique to Kiribati; other Pacific atoll countries such as Tuvalu, the Marshall Islands and Tokelau may benefit particularly from utilization of the approach outlined in this paper and the lessons learned from Kiribati’s experience in finalizing and implementing its NCCHAP.

## References

[1] (2012). Ministry of Finance and Economic Planning 2010.

[2] (2011). Climate Change in the Pacific: Scientific Assessment and New Research—Country Report for Kiribati.

[3] Aung T., Singh A., Prasad A. (2009). A study of sea-level changes in the Kiribati area for the last 16 years. Weather.

[4] McMichael T., Montgomery H., Costello A. (2012). Health risks, present and future, from global climate change. BMJ.

[5] Haines A., Kovats R.S., Campbell-Lendrum D., Corvalan C. (2006). Climate change and human health: Impacts, vulnerability and public health. Public Health.

[6] Singh S., Mushtaq U., Holm-Hansen C., Milan D., Cheung A., Watts N. (2011). The importance of climate change to health. Lancet.

[7] Patz J.A., Campbell-Lendrum D., Holloway T., Foley J.A. (2005). Impact of regional climate change on human health. Nature.

[8] Kovats R.S., Menne B., McMichael A.J., Corvalan C., Bertollini R. (2000). Climate Change and Human Health: Impact and Adaptation.

[9] Woodward A., Hales S., Weinstein P. (1998). Climate change and human health in the Asia Pacific region: Who will be most vulnerable?. Clim. Res..

[10] Lovell S.A. (2011). Health governance and the impact of climate change on Pacific small island developing states. IHDP Update.

[11] Singh R.B., Hales S., de Wet N., Raj R., Hearnden M., Weinstein P. (2001). The influence of climate variation and change on diarrheal disease in the Pacific Islands. Environ. Health Perspect..

[12] Ebi K.L., Lewis N.D., Corvalan C., Lucia S. (2006). Climate variability and change and their potential health effects in small island states: Information for adaptation planning in the health sector. Environ. Health Perspect..

[13] Spickett J.T., Katscherian D., McIver L. (2013). Health impacts of climate change in Vanuatu: An assessment and adaptation action plan. Glob. J. Health Sci..

[14] (2009). WHO and Secretariat for the Pacific Community Madang Commitment.

[15] (2007). Regional Framework for Action to Protect Human Health from Effects of Climate Change in the Asia-Pacific Region.

[16] (2007). National Adaptation Programme of Action (NAPA).

[17] Campbell-Lendrum D., Woodruff R. (2007). Climate Change: Quantifying the Health Impact at National and Local Levels. http://whqlibdoc.who.int/publications/2007/9789241595674_eng.pdf?ua=1.

[18] Ebi K.L., Kovats R.S., Menne B. (2006). An approach for assessing human health vulnerability and public health interventions to adapt to climate change. Environ. Health Perspect..

[19] Spickett J.T., Brown H.L., Katscherian D. (2011). Adaptation strategies for health impacts of climate change in Western Australia: Application of a health impact assessment framework. Environ. Impact Assess. Rev..

[20] Kovats S., Ebi K., Menne B. Methods of Assessing Human Health Vulnerability and Public Health Adaptation to Climate Change. http://www.euro.who.int/__data/assets/pdf_file/0009/91098/E81923.pdf.

[21] Prüss-Ustün A., Corvalán C. (2007). How much disease burden can be prevented by environmental interventions?. Epidemiology.

[22] (2011). Bank, World. Kiribati Adaptation Program—Phase III Project Appraisal Document.

[23] Cheng J.J., Berry P. (2013). Health co-benefits and risks of public health adaptation strategies to climate change: A review of current literature. Int. J. Public Health.

[24] Ganten D., Haines A., Souhami R. (2010). Health co-benefits of policies to tackle climate change. Lancet.

[25] Parker C.L. (2014). Health impacts of sea-level rise. Plan. Environ. Law.

[26] Hashizume M., Armstrong B., Hajat S., Wagatsuma Y., Faruque A.S.G., Hayashi T., Sack D.A. (2007). Association between climate variability and hospital visits for non-cholera diarrhoea in Bangladesh: Effects and vulnerable groups. Int. J. Epidemiol..

[27] Milojevic A., Armstrong B., Hashizume M., McAllister K., Faruque A., Yunus M., Streatfield P.K., Moji K., Wilkinson P. (2012). Health effects of flooding in rural Bangladesh. Epidemiology.

[28] Funari E., Manganelli M., Sinisi L. (2012). Impact of climate change on waterborne diseases. Ann. Ist. Super. Sanita.

[29] Cann K.F., Thomas D.R., Salmon R.L., Wyn-Jones A.P., Kay D. (2013). Extreme water-related weather events and waterborne disease. Epidemiol. Infect..

[30] Spini L., Adeel Z., Rosenberg M.W. (2011). The nexus of water and human health in the context of global changes. Curr. Opin. Environ. Sustain..

[31] Fewtrell L., Kaufmann R., Kay D. (2005). Water, sanitation, and hygiene interventions to reduce diarrhoea in less developed countries: A systematic review and meta-analysis. Lancet Infect..

[32] Gunnarsdottir M.J., Gardarsson S.M., Elliott M., Sigmundsdottir G., Bartram J. (2012). Benefits of water safety plans: Microbiology, compliance, and public health. Environ. Sci. Technol..

[33] Markandya A., Chiabai A. (2009). Valuing climate change impacts on human health: Empirical evidence from the literature. Int. J. Environ. Res. Public Health.

[34] El-Fadel M., Ghanimeh S., Maroun R., Alameddine I. (2012). Climate change and temperature rise: Implications on food- and water-borne diseases. Sci. Total Environ..

[35] Semenza J.C., Suk J.E., Estevez V., Ebi K.L., Lindgren E. (2012). Mapping climate change vulnerabilities to infectious diseases in europe. Environ. Health Perspect..

[36] Rose J.B., Epstein P.R., Lipp E.K., Sherman B.H., Bernard S.M., Patz J.A. (2001). Climate variability and change in the United States: Potential impacts on water- and foodborne diseases caused by microbiologic agents. Environ. Health Perspect..

[37] Chowell G., Cazelles B., Broutin H., Munayco C.V. (2011). The influence of geographic and climate factors on the timing of dengue epidemics in Perú, 1994–2008. BMC Infect. Dis..

[38] Thai K.T.D., Anders K.L. (2011). The role of climate variability and change in the transmission dynamics and geographic distribution of dengue. Exp. Biol. Med..

[39] Rosa-Freitas M.G., Schreiber K.V., Tsouris P., Weimann E.T.D.S., Luitgards-Moura J.F. (2006). Associations between dengue and combinations of weather factors in a city in the Brazilian Amazon. Rev. Panam. Salud Publica.

[40] Hunter P.R. (2003). Climate change and waterborne and vector-borne disease. J. Appl. Microbiol..

[41] Hales S., de Wet N., Maindonald J., Woodward A. (2002). Potential effect of population and climate changes on global distribution of dengue fever: An empirical model. Lancet.

[42] Guzman M.G., Halstead S.B., Artsob H., Buchy P., Farrar J., Gubler D.J., Hunsperger E., Kroeger A., Margolis H.S., Martínez E. (2010). Dengue: A continuing global threat. Nat. Rev. Microbiol..

[43] Weiss R.A., McMichael A.J. (2004). Social and environmental risk factors in the emergence of infectious diseases. Nat. Med..

[44] Lowe R., Bailey T.C., Stephenson D.B., Jupp T.E., Graham R.J., Barcellos C., Carvalho M.S. (2013). The development of an early warning system for climate-sensitive disease risk with a focus on dengue epidemics in southeast Brazil. Stat. Med..

[45] Chaves L.F., Pascual M. (2007). Comparing models for early warning systems of neglected tropical diseases. PLoS Negl. Trop. Dis..

[46] Yu H.-L., Yang S.J., Yen H.J., Christakos G. (2011). A spatio-temporal climate-based model of early dengue fever warning in southern Taiwan. Environ. Res..

[47] Paynter S., Ware R.S., Weinstein P., Williams G., Sly P.D. (2010). Childhood pneumonia: A neglected, climate-sensitive disease?. Lancet.

[48] Takaro T. (2013). Climate change and respiratory health: Current evidence and knowledge gaps. Expert Rev. Resp. Med..

[49] Ebi K.L. (2008). Adaptation costs for climate change-related cases of diarrhoeal disease, malnutrition, and malaria in 2030. Global Health.

[50] Friel S., Bowen K., Campbell-Lendrum D., Frumkin H., McMichael A.J., Rasanathan K. (2011). Climate change, noncommunicable diseases, and development: The relationships and common policy opportunities. Annu. Rev. Public Health.

[51] Kjellstrom T., McMichael A.J. (2013). Climate change threats to population health and well-being: The imperative of protective solutions that will last. Glob. Health Action.

[52] Tawatsupa B., Dear K., Kjellstrom T., Sleigh A. (2014). The association between temperature and mortality in tropical middle income Thailand from 1999 to 2008. Int. J. Biometeorol..

[53] Lundgren K., Kuklane K., Gao C., Holmér I. (2013). Effects of heat stress on working populations when facing climate change. Ind. Health.

[54] Hales S., Weinstein P., Woodward A. (1999). Ciguatera (fish poisoning), El Niño, and Pacific Sea surface temperatures. Ecosyst. Health.

[55] Skinner M.P., Brewer T.D., Johnstone R., Fleming L.E., Lewis R.J. (2011). Ciguatera fish poisoning in the Pacific Islands (1998 to 2008). Methods.

[56] Llewellyn L.E. (2010). Revisiting the association between sea surface temperature and the epidemiology of fish poisoning in the South Pacific: Reassessing the link between ciguatera and climate change. Toxicon.

[57] Berry H.L., Bowen K., Kjellstrom T. (2010). Climate change and mental health: A causal pathways framework. Int. J. Public Health.

[58] Albrecht G., Sartore G., Connor L., Higginbotham N., Freeman S., Kelly B., Stain H., Tonna A., Pollard G. (2007). Solastalgia: The distress caused by environmental change.

[59] Davidson J.R., McFarlane A.C. (2006). The extent and impact of mental health problems after disaster. J. Clin. Psychiat..

[60] Berry H., Bowen K., Kjellstrom T. (2010). Climate change and mental health—A causal pathways framework. Int. J. Public Health.

[61] Obrien L.V., Berry H.L., Coleman C., Hanigan I.C. (2014). Drought as a mental health exposure. Environ. Res..

[62] Mcmichael C., Barnett J., Mcmichael A.J. (2012). An ill wind? Climate change, migration, and health. Environ. Health Perspect..

[63] Heltberg R., Siegel P.B., Jorgensen S.L. (2009). Addressing human vulnerability to climate change: Toward a “no-regrets” approach. Glob. Environ. Chang..

[64] Wardekker J.A., de Jong A., van Bree L., Turkenburg W.C., van der Sluijs J.P. (2012). Health risks of climate change: An assessment of uncertainties and its implications for adaptation policies. Environ. Health.

[65] Patz J., Campbell-Lendrum D., Gibbs H., Woodruff R. (2008). Health impact assessment of global climate change: Expanding on comparative risk assessment approaches for policy making. Annu. Rev. Public Health.

[66] Chalabi Z., Kovats S. (2014). Tools for developing adaptation policy to protect human health. Mitig. Adapt. Strateg. Glob. Change.

[67] Randolph S.E. (2013). Is expert opinion enough? A critical assessment of the evidence for potential impacts of climate change on tick-borne diseases. Anim. Health Res. Rev..

